# Emotion socialization practices in Italian and Hong Kong-Chinese mothers

**DOI:** 10.1186/s40064-015-1550-1

**Published:** 2015-12-07

**Authors:** Caterina Fiorilli, Simona De Stasio, Carlo Di Chicchio, Siu Mui Chan

**Affiliations:** LUMSA University, P.zza delle Vaschette, 101, 00193 Rome, Italy; INVALSI, Via Borromini, 5, Frascati, Italy; Departmento of Psychocological Studies, The Hong Kong Institute of Education, 10 Lo Ping Road, Tai Po, New Territories, Hong Kong

**Keywords:** Emotion expression, Emotion socialization, Hong Kong-Chinese mothers, Italian mothers

## Abstract

**Background:**

Parents’ emotion socialization practices are their ways of handling their children’s emotional experiences in light of cultural expectations surrounding emotions. Such practices are thought to significantly affect children’s social adjustment. We compared the parenting practices of Italian and Hong Kong Chinese samples in an ethnological validation study of the maternal responses to children’s emotion scale (MRCES). Participants were 71 Italian mothers (M = 39.45) and 71 Hong Kong-Chinese mothers (M = 37.75) with children aged 6–9 years.

**Findings:**

The results confirmed the two-factor structure identified by the scale’s authors, namely coaching/emotion-encouraging and emotion dismissing approaches, respectively. Each of the two factors displayed satisfactory internal consistency. The Chinese mothers obtained higher scores than the Italian mothers on both subscales.

**Conclusions:**

Our findings suggested that parents’ emotion socialization practices for coping with children’s emotions received different degrees of emphasis and were underpinned by different meaning in the Hong Kong and Italian cultural groups. These cultural differences are discussed in relation to their effect on children’s socio-emotional development.

## Background

Parental emotion socialization plays a vital role in the quality of children’s early emotional competence (Brown and Dunn 1996; Eisenberg et al. 1998; Saarni 1999). In middle childhood, parents continue to shape the development of children’s emotional skills, although peers become more influential agents in the socialization process (Underwood and Hurley 1999). Parents’ emotion socialization practices are the ways in which they handle emotional experience, teach and talk to their children about emotional events, and initiate them in cultural expectations concerning emotions (Chan et al. 2009; Chang et al. 2003; Denham and Kochanoff 2002; Denham et al. 2002; Eisenberg et al. 1998; Fabes et al. 2002). Factors that help a cultural group to adjust to its environment are thus transmitted from generation to generation via socialization. These practices are thought to significantly affect children’s emotional development in areas such as their coping strategies (Eisenberg et al. 1998), emotion understanding (Bornstein et al. 2008; De Stasio et al. 2014; Eisenberg et al. 1998) and social adjustment (Eisenberg and Fabes 1994; Zeman et al. 2002). Eisenberg et al. (1998) proposed grouping parental emotion-related socialization practices into three types: (a) non-supportive *vs* supportive reactions to children’s experience and expression of emotion; (b) discussion of emotion; (c) parental expressiveness (Eisenberg et al. 1998). In this model, non-supportive reactions to emotion include parental practices such as punitive, dismissing and distressed reactions (Tao et al. 2010), whereas supportive reactions are those that validate children’s feelings. Discussion of emotions is viewed as positive when it provides a means of enhancing reflective problem-solving about emotions (Chan et al. 2009). Finally, parents’ own nonverbal and verbal expression of emotion may serve as a model for their children (Halberstadt et al. 1995). Halberstadt et al. (1995) distinguished between positive forms of expressiveness, characterized by warmth, openness and sensitivity, and negative ones, marked by anger and fear. With regard to parents’ contingent responses to their children’s emotions, these convey messages about cultural values surrounding the expression and regulation of emotion (Chan et al. 2009; Wu et al. 2002).

In recent years, an increasing number of comparative studies have examined emotion socialization practices across different cultural groups with a view to identifying shared *vs* different patterns (see Bornstein et al. 2008). Accordingly, the current study was designed to explore similarities and differences in Italian and Hong-Kong mothers’ responses to their children’s expression of emotions. To our knowledge, emotion socialization practices in these two cultural contexts have not yet been compared using a single validated scale and following recommended ethnological procedures.

### Cultural issues in emotion socialization practices

 A vast literature documents the influence of cultural factors on emotional experience, the expression of emotion and related behaviours, throughout child development (Bornstein et al. 2008; Cole et al. 2006; Eisenberg et al. 1998; Saarni 1999). It follows that any account of how children’s emotions are socialized will be incomplete if it does not examine the role of cultural environment.

 Numerous authors have proposed that cultural emotion socialization practices differ between collectivist and individualistic cultures (e.g., Eid and Diener 2001; Kitayama et al. 2000; Li et al. 2004; Markus and Kitayama 1991; Mesquita and Karasawa 2004; Schimmack et al. 2002; van Hemert et al. 2007). In collectivist cultures, typical of Oriental societies, the leading values are interdependence among individuals and group identity. Parents’ emotion socialization practices thus promote emotional self-regulation, limited expression of negative emotions and a high level of self-control on the part of their children, in the interest of maintaining social harmony and respecting the emotions of other group members (Mesquita and Frijda 1992).

Conversely, the educational priorities of parents in the individualist cultures associated with Western societies are to foster individual independence, personal assertion, and the achievement of personal goals (Hofstede 2001). Consequently, emotional expressiveness is encouraged, and intra-personal and subjective experience significantly contribute to defining individual feelings.

In line with this distinction, cultural ideals of emotion differ greatly between East Asian and Western culture. East Asians such as the Chinese tend to moderate emotional experience and expression (e.g., Markus and Kitayama 1991; Wu 1985). In Western culture on the other hand, emotion is often viewed as a spontaneous manifestation of personal experience rather than as related to the experience of others (Markus and Kitayama 1991). Furthermore, expressing oneself in Western culture is often viewed as beneficial for one’s mental and physical health (Butler et al. 2007; Gross and John 2003; John and Gross 2004).

According to Halberstadt and Lozada (2011), restraint in expressing positive affect seems more likely to occur in collectivist than in individualistic societies. More specifically, Chinese parents have been found to score higher on measures of “restrictive”, “controlling”, and “authoritarian” parenting than Western parents (Chao 2001; Kelley and Tseng 1992; Szeto et al. 2009). They also tend to emphasize the social environment rather than hereditary factors in explaining individual differences among children (Ho and Kang 1984). Therefore, Chinese parents tend to be very involved in their children’s upbringing. The main role attributed them is that of the teacher (Kelley and Tseng 1992) and they see discipline as a key parental child-rearing tool (Chen and Luster 2002; Miller et al. 2002).

In Italian culture on the other hand, parents encourage emotional expressiveness in interpersonal interaction, promote social competence during early childhood, and consider expressions of inhibition, caution, or withdrawal as indicative of poor social skills (Chen et al. 2000; Edwards et al. 1996). The importance attributed to social intelligence and emotional experience underpins a cultural model centred on the infant’s ability to build significant relationships from the earliest months of life. To this end, the developing infant is expected to display emotional closeness, involving expressiveness, liveliness and closeness to other people (the “sociable” child), as well as emotional security. Overall, a composite picture emerges in which socio-emotional closeness and emotional security form a core developmental goal for Italian children.

Norms regarding emotion vary with culture, as do the elicitation, regulation and manifestation of emotion (Eid and Diener 2001; Markus and Kitayama 1991), while the ability to produce culturally appropriate expressions of emotion is a key component of social competence (e.g., Eisenberg et al. 1996; Halberstadt and Lozada 2011; Saarni 1999).

Nevertheless, previous research also found significant differences in emotion socialization practices within the broad cultural groups we have discussed up to now (cfr., Hofstede 2001). In reality, the degree to which Western and Eastern parents believe that children can be in charge of their own emotional lives and that parents need to guide their children and teach them about emotions, vary as a function of class and ethnicity (Craig et al. 2010). For example, in relation to the power distance theory (Hofstede 2001; Halberstadt and Lozada 2011), Italian mothers seem more emotionally sensitive than American mothers when interacting with their infants. Furthermore, comparisons of American and Italian mothers, and Japanese and American mothers, belonging to collectivist and individualist societies respectively, have shown greater similarities between American and Japanese parenting practices, than similarities between parents from societies within the same broad cultural group. Specifically, American and Japanese mothers expect earlier emotional competence from their children than their counterparts from other countries (Goodnow et al. 1984).

Up to now, however, cross-cultural studies comparing East and West, have not analysed parental practices in Italy and Hong Kong. Given that cultural comparisons are an excellent means of determining which aspects of development are universal and which vary as a function of culture, the current study aimed to contribute cross-cultural evidence from a previously unexplored perspective.

### The present study

Based on the literature reviewed above, we assumed that the mothers in the Hong Kong and Italian cultural groups would display different patterns of response to their children’s emotions on the basis that certain aspects of parenting may be valued differently, attributed with different meanings, or enacted differently in the two contexts. The main goal of the study was to compare Hong Kong-Chinese and Italian mothers’ responses to their children’s emotional expression using an invariant (comparable) measure of parental emotion socialization practices. First, we analysed the psychometric properties of the Italian version of the maternal responses to children’s emotion scale (MRCES) which had previously been tested with a Chinese-Honk Kong sample. We used exploratory and confirmatory factorial analysis to verify whether Chinese and Italian groups’ responses to the MRCES shared the same factor structure. A previous study using the MRCES with a sample of Hong Kong mothers had found a two-factor solution with robust goodness of fit to the data (Chan et al. 2009). The questionnaire’s eight items loaded on the two factors as follows: Factor 1 was labelled the coaching-emotion encouraging approach and was composed of items assessing: emotion-focused, problem-focused, reflection-enhancing, training, and expression-encouraging responses; Factor 2 was termed the emotion dismissing approach and comprised items evaluating: punitive, minimizing and distressed responses to children’s expression of emotions. We expected to find a similar structural-factor solution in the two cultural settings investigated in the current research.

Second, we explored whether specific cultural emphases would be identified by analysing differences in the mean MRCES responses provided by Hong Kong and Italian mothers of school-age children. Because there has been no other comparison of emotion socialization practices in Italian and Hong-Kong cultures and due to the heterogeneous findings on parental practices within and between Western and East societies we did not formulate any a priori hypotheses about cultural similarities or differences in the mean level of mothers’ coaching/emotion encouraging or emotion dismissing responses to their children’s emotional expression.

## Methods

### Participants

Participants were selected from two larger samples in both countries, including 71 Italian mothers and 71 Hong Kong-Chinese mothers (N = 142). Criteria for inclusion were based on characteristics of children (e.g., IQ within the average range or higher, free from chronic medical conditions or severe psychosis) and family structure (e.g., consistent mother figure; no major structural changes, such as divorce, within the past year). The two sub-samples were homogeneous in terms of children’s gender (Chinese group: 33 female and 38 males; Italian group: 36 female, 35 male; Chi^2^ = .614; df = 1; p = .433) and age (Chinese group: 43 children aged 6–7 years, and 28 children aged 8–9 years; Italian group: 38 children aged 6–7 years, 33 children aged 8–9 years; Chi^2^ = .397; df = 1; p = .529). Based on the original contribution by Chan et al. (2009) we recruited mothers of school-aged children. The authors reported that in the first years of elementary school there is an increase of both children’s emotion coping skills and their social competence, thus suggesting a meaningful context for studying emotion socialization. For these reasons, the present study focused on the emotion socialization patterns of parents of children in the first and second years of school.

Chinese and Italian mothers aged 30–44 years (respectively M = 37.75, 39.45). Finally, all mothers were high school educated. In both cultural settings mothers were recruited by means of school contact with a participation rate above 75 %.

### Instruments

Parental practices in response to children’s emotion expression were measured by means of the maternal responses to children’s emotion scale (MRCES; Chan et al. 2009), which consists of 12 scenarios (items) describing typical situations in which children experience strong emotions (e.g., a favourite toy being broken by their mother, being scared after watching a TV program) or express emotions in inappropriate ways (e.g., showing disappointment on the spot when receiving a present he or she does not like, or continuing to boast that he or she has beaten a friend in a game). For each scenario, mothers were asked to rate from 1 (*never*) to 6 (*always*) how likely it was for them to react using each of the following eight types of responses: (1) emotion-focused; (2) problem-focused; (3) reflection-enhancing; (4) training; (5) expression-encouraging; (6) punitive; (7) minimization; (8) distress. The scale was forward- and back-translated from Chinese to English, then English to Italian and finally from Italian to English by two independent and fluent translators assisted by the authors in order to overcome incoherence. Then, professionals and bilingual mothers who lived in Italy and were not participants in the study were interviewed regarding the cultural validity of items in the instruments. Finally, pilot testing was undertaken to ensure that the instruments were comprehensible and ethnographically valid. By adopting this procedure, similarity in meaning of the items was assured, taking into account both cultures. Scores for each responses, either positive or negative, were computed by averaging the ratings for each of the 8 strategies, across the 12 scenarios, keeping the scale unit from 1 to 6.

## Results

The analyses were conducted in several steps. First, two exploratory Principal Components Analyses (at the item level and scale level) were performed in order to discover the factor structure of the MRCES in the combined sample. Second, several Confirmatory Factor Analyses were conducted in order to test the factor structure of the MRCES within each group (Chinese and Italian) and by means of a multigroup analysis, to test structural invariance with different levels of constraints. Finally a Multivariate Analysis of Variance (MANOVA) was conducted in order to test whether the Chinese and Italian mothers showed significant mean differences both on the overall indices (factor scores) and on mothers’ endorsement of individual strategies.

### Exploratory principal components analyses (PCA)

Exploratory PCA at the item level showed that each scale was unidimension with high internal consistency (Cronbach’s alpha larger than .80). Further, Exploratory PCA at the scale level confirmed the two-factor solution with non-correlated factors, as shown in Chan and coll.’s research (2009). Factor 1 (coaching-emotion encouraging approach) included experience enhancing responses, problem focusing responses, reflection enhancing responses, emotion focusing responses and training responses; Factor 2 (emotion dismissing approach) included punitive, distress and minimization responses.

### Results of separate confirmatory factor analyses (CFA)

One of the main goals of the study was to examine whether the Chinese group and the Italian group shared the same factor structure. As a first step we conducted a separate CFA for each group. For the Chinese group, the Chi square statistic was significant (Chi square = 33.13; df = 19; p < .05), suggesting that the factor structure differed from that found in the combined sample. However, other indices showed an acceptable fit of the tested model. For example, the Non Centrality Parameter was well within the 90 % confidence interval (NCP = 14.12; CI 1.91–34.16). Similarly, although RMSEA showed an absolute value higher the .08 cut-off, it was within the 90 % confidence interval, with a non-significant test of closeness-of-fit (RMSEA = .11; CI .041–.17; p-close = .07). The same figure emerged looking at the Expected Cross-validation Index (ECVI = 1.14; CI .93–1.48; ECVI for saturated model = 1.22); the Comparative Fit Index (CFI = .93); and the Incremental Fit Index (IFI = .94). On the other hand, GFI and AGFI were below the .90 cut-off, indicating that the tested model reproduced poorly the observed covariances.

For the Italian group the Chi square statistic was non-significant (Chi square = 27.58; df = 19; p < .10) and the other fit indices showed a more acceptable fit of the model in comparison with the Chinese group. The Non-Centrality Parameter was within the 90 % confidence interval (NCP = 8.58; CI .00–.38), as well as RMSEA showed an absolute value of .08, within the 90 % confidence interval, with a non-significant test of close-fit (RMSEA = .08; CI .00–.14; p-close = .22). Other fit indices as Expected Cross-validation Index (ECVI = .88; CI .76–1.14; ECVI for saturated model = 1.03); Comparative Fit Index (CFI = .98) and Incremental Fit Index (IFI = .98) showed a better fit for the Italian group. The GFI was .90 while AGFI was below the .90 cut-off.

### Results of multigroup confirmatory factory analyses

The subsequent multigroup analyses were performed with the objective to cross-validate the factor structure in the two groups. The analyses were conducted allowing several degrees of invariance: first, a multigroup analysis was performed without any invariance constraint. The aim was to test whether in the Chinese and Italian groups the same factor structure emerged (baseline model). Second, the factor loadings invariance was tested; finally the invariance of the error variance was tested along with the factor loadings invariance (Table 1).

**Table 1:** Goodness-of-fitindexes from multigroup analysis of maternal responses to children’s emotion scale (MRCES)

Models	χ^2^ (df)	RMSEA	CFI	χ_diff_^2^ (df)
Within-group models				
Chinese	33.12* (19)	.11	.93	
Italian	27.58 (19)	.08	.98	
Multigroup model				
No-constraints	60.70* (38)	.09	.96	
Factor loadings invariance	66.91* (45)	.09	.96	
Difference between model 1 and model 2				6.21 (7)
Factor loadings and measurement error invariance	90.51* (53)	.10	.92	23.6* (8)
Difference between model 2 and model 3				

*RMSEA* root mean square error of approximation, *CFI* comparative fit index

* *p* < .05

With regards to the baseline model (no-constraints), the results showed that, overall, the two groups shared a common factor structure. In fact, although the Chi square statistic was significant (Chi square = 60.70; df = 38; p < .05), NCP and RMSEA were within the 90 % confidence interval, showing an acceptable fit (NCP = 22.70; CI 5.32–48.00; RMSEA = .09; CI .05–.14; p-close = .60). Similarly, CFI and IFI showed a value of .96.

There were similar results for the model that tested factor loadings invariance. The Chi square statistic was significant (Chi square = 66.91; df = 45; p < .05), while NCP, RMSEA, CFI and IFI showed the same values as for the previous model. In order to test whether the increase of the Chi square was significant, thus rejecting the hypothesis of factor loadings invariance, the baseline Chi square was subtracted from the factor loadings invariance model Chi square, and the same was done with their degrees of freedom. The distribution of the resulting value is a Chi square distribution with degrees of freedom equal to the difference of the degrees of freedom of the two models. The results of this computation showed that the increase of the Chi square statistic was not significant (ΔChi square = 6.21; df = 7; p > .05), meaning that the Chinese group and the Italian group shared the same factor structure measured on the same scale.

Finally, the equality of measurement errors was tested between the two groups. The results of this analysis showed a significant decrease of the model fit compared with the previous one.

### Differences between Chinese and Italian samples

After the invariance of factor loadings was supported by the results of the multigroup CFA, the difference between the Chinese and Italian samples was assessed in terms of each factor. A multivariate ANOVA was conducted in order to evaluate this difference, and the results showed that the Chinese and Italian groups were different at the multivariate level (Wilks’ Lambda = .831; F(2,138) = 14.057; p < .001; partial η^2^ = .17) as well as the univariate level (coaching-emotion encouraging approach: F(1,139) = 18.320; p < .001; partial η^2^ = .12; emotion dismissing approach: F(1,139) = 14.467; p < .001; partial η^2^ = .09). In particular, the Chinese group had an average score on both factors that was significantly higher than that of the Italian group (Table 2).

**Table 2:** Mean scores on MRCES factors by group membership

Variables	M (SD)	95 % CI	
		LL	UL
Emotion dismissing			
Chinese group^a^	3.5_b_ (.60)	3.3	3.7
Italian group^c^	3.1_d_ (.74)	2.9	3.2
Coaching			
Chinese group	4.6_e_ (.37)	4.5	4.7
Italian group	4.2_f_ (.54)	4.1	4.3

Means that share the same subscript do not differ significantly

*CI* confidence interval, *LL* lower limit, *UL* upper limit

^a^ *n* = 70, ^c^ *n* = 71

The differences between the Chinese and the Italian samples on maternal strategies were analysed by a Multivariate Analysis of Variance (MANOVA). The first analysis examined group differences on the strategies that made up the coaching-emotion encouraging approach component from the previous Principal Component Analysis. The second analysis examined the strategies that made up the emotion dismissing approach component from the same PCA.

With respect to the emotion coaching strategies, MANOVA results pointed out a statistically significant multivariate effect of group membership (Wilks’ Lambda = .614; F_5,131_ = 16.47; p = .000; partial η^2^ = .39). At the univariate level, statistically significant differences between groups emerged on: *Expression Encouraging* (F_1,135_ = 22.23; p = .000; partial η^2^ = .14), *Problem Focused* (F_1,135_ = 25.36; p = .000; partial η^2^ = .16), *Emotion Focused* (F_1,135_ = 15.82; p = .000; partial η^2^ = .11), and *Training* (F_1,135_ = 10.52; p = .001; partial η^2^ p = .07). No differences emerged for the *Reflection Enhancing* strategy. Observing the group means, the Chinese mothers reported employing these positive strategies more often than Italian mothers, with the exception of reflection enhancing, which was used by both groups to the same degree (Table 3).

**Table 3:** Mean scores on MRCES coaching strategies by group membership

Variables	M (SD)	95 % CI	
		LL	UL
Expression encouraging			
Chinese group^a^	4.3_b_ (.55)	4.1	4.5
Italian group^c^	3.8_d_ (.76)	3.6	3.9
Problem focused			
Chinese group	4.5_e_ (.07)	4.4	4.6
Italian group	4.0_f_ (.07)	3.9	4.1
Reflection enhancing			
Chinese group	4.8 _g_ (.08)	4.6	4.9
Italian group	4.8 _g_ (.07)	4.6	4.9
Emotion focused			
Chinese group	4.5 _h_ (.08)	4.3	4.6
Italian group	4.1_i_ (.08)	3.9	4.2
Training responses			
Chinese group	4.9_j_ (.06)	4.8	5.1
Italian group	4.7 _k_ (.06)	4.5	4.8

Means that share the same subscript do not differ significantly

*CI* confidence interval, *LL* lower limit, *UL* upper limit

^a^ *n* = 66, ^c^ *n* = 71

As far as concerned the dismissing strategies, MANOVA results pointed out a statistically significant multivariate effect of group membership (Wilks’ Lambda = .795; F_3,129_ = 11.10; p = .000; partial η^2^ = .21). At the univariate level, statistically significant differences between groups emerged on *Minimization* (F_1,131_ = 12.96; p = .000; partial η^2^ = .09), and *Distress* (F_1,131_ = 17.07; p = .000; partial η^2^ = .12). No differences emerged for the *Punitive* strategy. Observing the group means, the Chinese mothers reported employing negative strategies more than Italian mothers, with the exception of punitive strategies, which were shown to the same degree in both groups (Table 4).

**Table 4:** Mean scores on MRCES emotion dismissing strategies by group membership

Variables	M (SD)	95 % CI	
		LL	UL
Minimization			
Chinese group^a^	3.7_b_ (.59)	3.6	3.9
Italian group^c^	3.3_d_ (.74)	3.2	3.5
Punitive			
Chinese group	3.3_e_ (.70)	3.1	3.5
Italian group	3.1_e_ (.86)	2.9	3.3
Distress			
Chinese group	3.4_f_ (.77)	3.1	3.6
Italian group	2.8 _g_ (.84)	2.6	3.0

Means that share the same subscript do not differ significantly

*CI* confidence interval, *LL* lower limit, *UL* upper limit

^a^ *n* = 62, ^c^ *n* = 71

## Discussion

A major contribution of this research is that a model of maternal responses to children’s expression of emotion empirically developed in the Hong Kong Chinese cultural context was found to be applicable to Italian mothers. This indicates that constructs representing maternal responses that are highly valued in China are measurable in Italy. Such findings reinforce the importance of using ecologically valid frameworks and functionally equivalent measures in cross-cultural studies (e.g., Little 1997). Comparative analysis of participants’ mean scores suggested that Hong Kong Chinese and Italian mothers significantly differed in relation to both types of maternal response to children’s emotion evaluated in the current study, namely the coaching-emotion encouraging approach and the emotion dismissing approach.

Specifically, Chinese mothers reported both types of responses more often than Italian mothers. Interestingly, no differences were found between the two cultural settings with regard to mothers’ specific use of reflection-enhancing responses (part of the overall coaching-emotion encouraging approach) or punitive responses (part of the emotion dismissing approach). Furthermore, the differences between the Chinese and the Italian mothers did not appear to be affected by sample composition. In fact, the distribution of children’s gender and age was similar across the two groups. The main findings are discussed further in the following section.

### Similarities and differences between Hong Kong-Chinese and Italian Mothers

Chinese mothers perceived themselves as responding to their children’s emotion expressions with coaching strategies more frequently than Italian mothers. The coaching approach, which involves maternal responses that may be emotion-focused (e.g., Distract his/her attention by turning on his/her favourite cartoon), problem-focused (e.g., Suggest that he/she can talk it over with the teacher), training (e.g., Tell my child that he/she should not behave so rudely because the teacher must have had reasons for giving a punishment), and expression-encouraging (e.g., Tell my child that I can see he/she is very angry), aims to teach children how to regulate and express their emotions appropriately. The high prevalence of these strategies among Chinese mothers is consistent with previous research. Specifically, Wu (1985) noted that Chinese parents are prone to be directive in their attempts to control and correct children’s behaviour, especially in terms of pushing them to engage in academic pursuits (Lin and Fu 1990). Maternal involvement and directiveness may reflect Chinese cultural beliefs that young children are incapable of understanding and making decisions that are in their own best interest. Consistently with this perspective, Chan et al. (2009) found a sample of Hong Kong-Chinese mothers to display higher levels of training and reflection-enhancing responses to their children’s emotion expression than any other emotion socialization response. This may reflect Hong Kong mothers’ preference to teach their children appropriate display rules by discussing the causes and consequences of expressing emotions with them or by explaining the moral reasons behind the rules. According to Wu and colleagues. (2002), the use of reasoning by Chinese parents may be partly influenced by Confucian traditions, in which children are encouraged to be sensitive, modest, and cooperative or “well-behaved”.

In light of the above, it seems plausible that the coaching/emotion encouraging approach should have been frequently adopted by the Hong Kong-Chinese mothers in the current study. However, further reflection is required as to why this type of response should be more frequent among Chinese mothers than among Italian mothers. Previous research has shown that Italian parents principally concentrate their energies on their affective relationship with their children, encouraging them to express their emotions; moreover, Italian mothers’ social representations of caregiving practices are predominantly characterized by affective and warm elements as opposed to objective and instrumental ones (Molinari and Speltini 1997). Earlier cross-cultural findings also suggested that, compared to US Mothers, Italian mothers display higher levels of social/affective behaviours and more openly express affection for their children (Hsu and Lavelli 2005). These same kinds of parental emotional competence are among the maternal responses assessed as part of the coaching-emotion encouraging approach in the current study.

In interpreting our findings, it is critical to bear in mind that “average” differences can be misleading, because there is almost always considerable overlap between comparison groups in areas of parental practice. Moreover, cultural and methodological issues may have contributed to differences in scores between groups, including cultural differences in perception, methodological issues with understanding items, or different standards for mastery.

With regard to the dismissing approach, Italian mothers revealed a significantly lower tendency to adopt non-supportive reactions than did Chinese mothers. The MRCES instrument measures this kind of strategy in terms of punitive responses (e.g., Scold him/her for having temper tantrum and being angry with teacher), minimization (e.g., Tell my child he/she is overreacting), and displays of distress in response to children’s expression of emotions (e.g., I would feel very angry). Research focused on Chinese parents’ goals in socializing their children’s emotions has suggested that Chinese parents do not view the dismissing approach in a negative light. Specifically, parental coaching and dismissing approaches to children’s emotion socialization are both embedded in the concept of “training” (Wu et al. 2002). Specifically, because “training” children is viewed as a key parental responsibility, parents report using both inductive and power-assertive disciplinary techniques to “train,” or “guan” their children (Lin 1999; Xu et al. 2005). In Xu’s study of family socialization and kindergarten children’s personality characteristics in China, parental control was significantly correlated with the use of reasoning and inductive strategies. Lin (1999) studied maternal parenting behaviour in Taiwan, pointing out that one-way discipline strategies (e.g., demanding, punishing) and two-way strategies (e.g., discussing) may both be adopted and share the same meaning in Chinese culture. It seems that Chinese parents set rules and restrictions for their children, but they also “reason” with their children, instead of just saying, “Do it because I say so”. However, given that young children lack self-control and continuously put the limits defined by their parents to the test, reasoning will not work in all situations. Therefore, Chinese parents believe that mild verbal or physical punishment may be used when the child continues to disobey his parents.

In the current study, the relatively lower adoption among Italian mothers of punitive, dismissing and distressed responses to their children’s emotion expression demands cautious interpretation as well as further study. Notably, in other studies, Italian mothers displayed *higher* rates of punitiveness than Canadian and French mothers across all age groups of children (Kane 2005), as well a significant level of normativeness and physical discipline with their children compared to, among others, Chinese mothers (Lansford et al. 2005). Nevertheless, it is important to take into account the mean age of the children to whom the mothers are referring in their responses. Furthermore, the low usage of the dismissing approach among Italian mothers recorded in the current study may be an underestimate, because Italian parents may be reluctant to self-report punitive, dismissive and distressed responses, given that such practices are not seen as positively in Italy as they might be in China. In general, this broad category of socialization practices meets a particularly low level of cultural approval in Western society (Halpenny et al. 2010).

Finally, aside from cultural differences, our findings are surprising given that emotion dismissing and coaching-emotion encouraging styles are conceptualized as disparate approaches to emotion socialization; thus, one would not expect emotion dismissing to be accompanied by high levels of emotion coaching (Gottman et al. 1996). Despite being conceptually in contrast with one another, positive correlations between emotion coaching and emotion dismissing have been found in other work (Duncombe et al. 2012; Havighurst et al. 2009; Wilson et al. 2012). Furthermore, in the parents’ of children in middle childhood, observed emotion coaching has been shown to moderate the effects of observed emotion dismissal on children’s emotionality and internalizing problems; in other words, parents’ engagement in emotion coaching buffered children from the negative effects of parental emotion dismissal (Lunkenheimer et al. 2007). In brief, parents may engage in both emotion coaching and emotion dismissing. In line with Miller et al. (2015) we may therefore interpret our findings as suggesting that parents who attend to emotion socialization use a variety of approaches depending on contextual demands. Future research could focus on how parents flexibly adapt their specific emotion socialization efforts to fit the situational context—for example, choosing when to coach emotions to scaffold a child’s problem solving, or when to dismiss emotions to help a child move on, within the framework of their overall emotion socialization style.

## Limitations and future research

The current study provides evidence of invariant dimensions of emotion socialization practices across two cultural groups, information that is helpful in validating the MRCES. However, some doubts remain about the differences between Italian and Hong Kong mothers found in this study. First, cultural differences in mothers’ responses may vary depending on what emotion is expressed by the child. For example, research has revealed large cultural differences regarding mothers’ treatment of their children’s anger and sadness, while few differences emerged across groups with respect to children’s physical pain (see Lansford et al. 2005). Next, it remains unclear whether mothers’ socialization practices vary with respect to their child’s gender. Because gender *roles* vary widely across different cultural contexts, future studies should systematically examine the contribution of parent and child gender to emotion socialization across cultures.

With regard to methodological aspects, we used only one self-report measure of emotion socialization and did not include any direct assessment of the social acceptability of child. Moreover, in the future, it will be of interest to analyse the same variables in a larger sample of mothers.

Finally, similar parental behaviours in different cultural contexts often involve very different parental motivations and explanatory systems, and are contextualized in different kinds of parent–child relationships. Consequently, parents’ emotional socialization practices may be understood and interpreted differently by children, and may be associated with different child outcomes, depending on the cultural context.
